# Appropriate PD-L1 Cutoff Value for Gastric Cancer Immunotherapy: A Systematic Review and Meta-Analysis

**DOI:** 10.3389/fonc.2021.646355

**Published:** 2021-09-01

**Authors:** Tong Xie, Zhening Zhang, Xiaotian Zhang, Changsong Qi, Lin Shen, Zhi Peng

**Affiliations:** Department of Gastrointestinal Oncology, Key Laboratory of Carcinogenesis and Translational Research (Ministry of Education), Peking University Cancer Hospital & Institute, Beijing, China

**Keywords:** immunotherapy, chemotherapy, PD-L1, cutoff value, gastric cancer

## Abstract

**Background:**

Immunotherapy dramatically changed the treatment landscape of gastric cancer in recent years. PD-L1 expression was proposed as a biomarker; however, the treatment strategy according to PD-L1 is still uncertain. Here, we aimed to find the appropriate cutoff value of PD-L1 expression for gastric cancer immunotherapy.

**Methods:**

We did a systematic electronic research of prospective clinical trials of gastric cancer immunotherapy across databases. Studies that provided subgroup analysis results stratified by PD-L1 expression were included. Objective response rate (ORR), disease control rate (DCR), hazard ratio (HR), and 95% confidential interval (CI) of progression-free survival (PFS) and overall survival (OS) at different PD-L1 cutoff values were extracted.

**Results:**

Twelve studies and 6,488 patients in total were finally included for pooled analysis. ORR in allover, PD-L1-negative, combined positive score (CPS) ≥1, CPS ≥5, and CPS ≥10 population was 10%, 3%, 13%, 20%, and 23%, respectively. Immune checkpoint inhibitor (ICI) monotherapy failed to show survival advantage in allover and PD-L1-negative patients. Single-agent ICI therapy prolonged OS (HR = 0.84, 95% CI: 0.74–0.96) but not PFS (HR = 1.38, 95% CI: 0.91–2.09) in PD-L1 CPS ≥1 patients. For combined immunotherapy, ORR in allover, PD-L1-negative, CPS ≥1, CPS ≥5, and CPS ≥10 population was 64%, 57%, 48%, 60%, and 58%, respectively. Allover population could gain survival benefit from combined immunotherapy based on the results from Checkmate-649. OS (HR = 0.81, 95% CI: 0.71–0.92) and PFS (HR = 0.77, 95% CI: 0.69–0.86) were significantly prolonged in PD-L1 CPS ≥1 patients receiving combined immunotherapy.

**Conclusion:**

Efficacy and survival advantages improved with PD-L1 CPS. CPS ≥1 was the cutoff value for ICI monotherapy to gain survival benefit. Combined immunotherapy prolonged PFS and OS in allover population but needs further study to confirm it.

## Introduction

Programmed death protein-1/ligand 1 (PD-1/PD-L1) inhibitors profoundly changed the treatment landscape of gastric cancer in recent years. However, due to a relatively low response rate, especially for single-agent immunotherapy, finding a dependable biomarker has become a spotlight in this field.

PD-L1 expression has been proposed as one of the pan-cancer biomarkers for immunotherapy. Combined positive score (CPS) and tumor proportional score (TPS) were proposed for PD-L1 assessment. As for gastric cancer, CPS was shown to be a more sensitive prognostic biomarker than TPS and, thus, was more widely used (1). In KEYNOTE-059, pembrolizumab exhibited favorable efficacy in gastric cancer, especially in PD-L1-positive patients, with an objective response rate (ORR) of 15.5% and duration of response (DOR) of 16.3 months (2). Owing to the results, pembrolizumab was approved for PD-L1-positive gastric cancer patients in second- or later-line treatment by the Food and Drug Administration (FDA). However, the predictive value of PD-L1 expression in gastric cancer was challenged by other clinical trials. ATTRACTION-2 demonstrated that nivolumab was superior to placebo regardless of the expression of PD-L1; PD-L1-negative gastric cancer patients could also benefit from immunotherapy (3). On the other hand, although pembrolizumab failed to show superiority to paclitaxel in second-line gastric cancer treatment in KEYNOTE-061, post-hoc analysis revealed that the treatment effect was greater in patients with a PD-L1 CPS ≥10 than CPS ≥1 (4). Also, in KEYNOTE-062, despite the failure of pembrolizumab in patients with PD-L1 CPS ≥1, a positive result was noticed in the PD-L1 CPS ≥10 population (5). Whether we should select patients according to PD-L1 expression and the possible PD-L1 expression cutoff value for our decision is of great importance to clinical practice, but the question remains unanswered.

The question was not only for single-agent immunotherapy but also for combined regimens. Immunotherapy plus chemotherapy has been tested in several clinical trials in recent years. The results seemed inconsistent, and the value of PD-L1 expression was also in doubt. KEYNOTE-062 reported a negative result of pembrolizumab in the first-line treatment of gastric cancer in PD-L1 CPS ≥1 population (5). However, in 2020 ESMO congress, Checkmate-649 reported positive results of first-line nivolumab plus chemotherapy compared with chemotherapy in gastric cancer. The success of nivolumab plus chemotherapy suited not only the PD-L1 CPS ≥5 or higher patients but also all the intention to treatment (ITT) population (6). Apart from pembrolizumab or nivolumab, other PD-1/PD-L1 inhibitors also reported results in gastric cancer (7). The utility of immunotherapy plus chemotherapy in gastric cancer has been justified; however, the target population and the role of PD-L1 expression in patient selection and management still need further investigation, which may differ from single-agent immunotherapy.

The value of PD-L1 expression in predicting gastric cancer immunotherapy is still not explicit. Here, we aimed to summarize the outcomes of current gastric cancer immunotherapy clinical trials and find the appropriate cutoff value of PD-L1 for clinical practice.

## Methods

### Searching Strategy and Criteria

Systemic search was conducted across databases in PubMed, Embase, Web of Science, and Cochrane Library in Oct 2020 in accordance with the Preferred Reporting Items for Systematic Review and Meta-Analyses (PRISMA) guidelines. Meeting abstracts published in the European Society for Medical Oncology (ESMO), the American Society of Clinical Oncology (ASCO), and ASCO-GI were also included in our searching scope. TX and ZZ screened the studies independently. Discrepancies were discussed and solved by supervisor LS. “Gastric cancer”, “PD-1”, “PD-L1”, “immunotherapy”, and the exact names of PD-1/PD-L1 inhibitors were also included in our searching frame such as pembrolizumab, nivolumab, and avelumab. Medical Subject Headings (MeSH) terms and related free text terms were also used. Studies were included if they met the following inclusion criteria: 1) randomized or non-randomized clinical trials that reported the efficacy or survival outcomes of PD-1/PD-L1 inhibitors or immune checkpoint inhibitor (ICI) plus chemotherapy for gastric cancer; and 2) studies that provided the results stratified by PD-L1 CPS. Retrospective study or studies that did not use English were excluded.

### Data Extraction

Articles, meeting abstracts, and matched supplementary materials were carefully read and examined. Clinical trials using a multi-cohort design were divided into individual arms for data extraction. Basic information of each study including study name, interventions, publication year, sample size, phase, and treatment line were documented. ORR, disease control rate (DCR), hazard ratio (HR), and 95% confidential interval (CI) for progression-free survival (PFS) and overall survival (OS) were extracted.

### Quality Assessment

For included randomized clinical trials (RCTs), the *Cochrane Handbook for Systematic Reviews of Interventions* was used for quality assessment, and it included allocation concealment, random sequence generation, blinding of participants, blinding of outcome assessment, incomplete outcome data, selective reporting, and other biases. We defined “+” as low risk of bias, “−” as high risk of bias, and “?” as insufficient for making precise judgment. Two independent authors (TX and ZZ) evaluated the bias assessment, and disagreements were resolved by supervisor LS.

### Statistical Analysis

For binary variables, such as ORR and DCR, events and sample size were used for analysis. We pooled the ORR and DCR at different PD-L1 cutoff values and by different interventions such as monotherapy or combined therapy. As for survival variants, logarithm of HR (logHR) and the standard error (SE) were calculated and then used for meta-analysis. Heterogeneity among studies was explored using the *I*2 test, and a *p*-value for heterogeneity was calculated. Random-effects model was used if high heterogeneity was noticed; otherwise, fixed-effects model was used. *p*-Value <0.05 was considered to indicate statistical significance. We used R version 3.6.3 to perform the meta-analysis.

## Results

### Research Results

**Figure 1:**
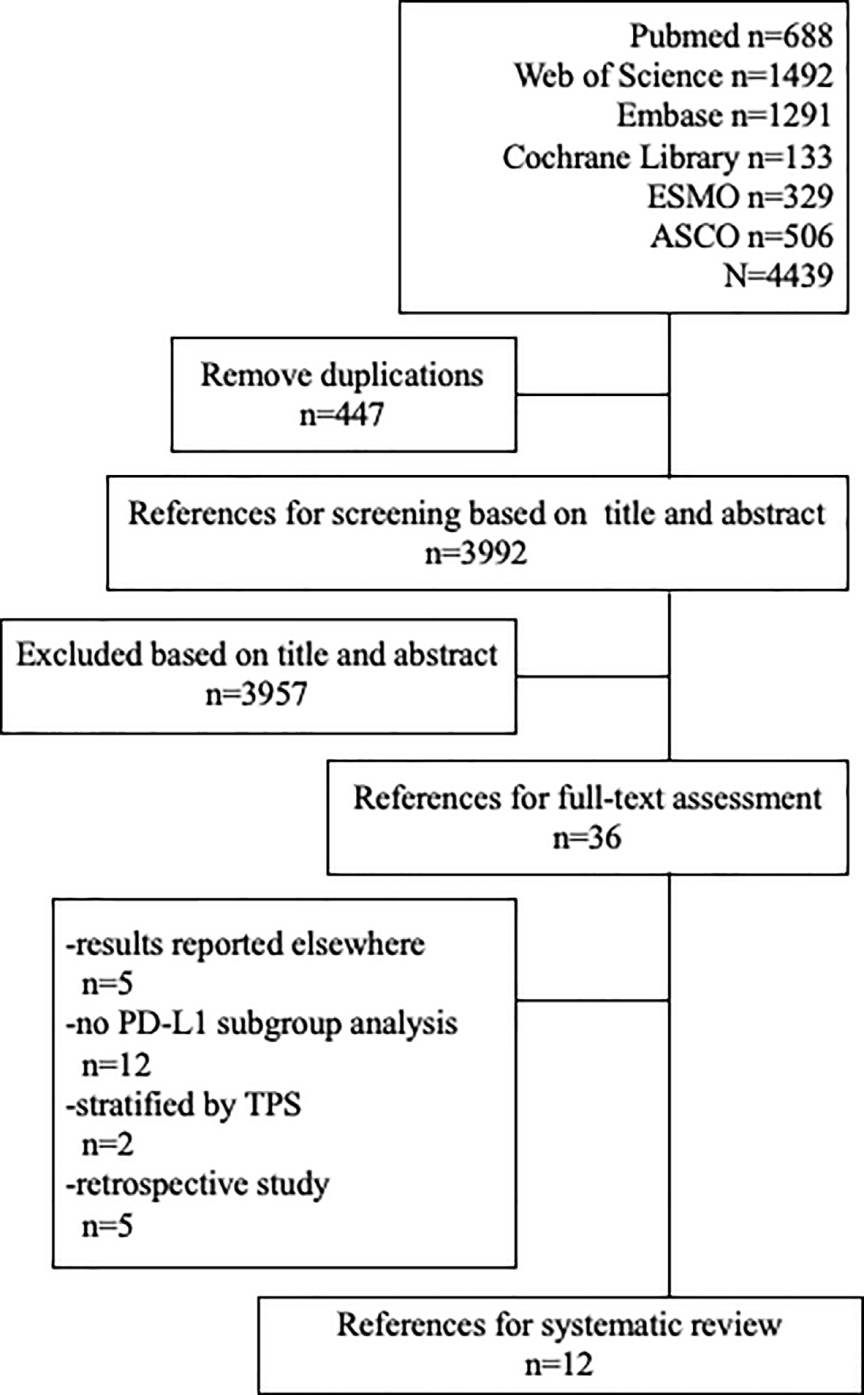
The diagram of searching process.

**Figure 2:**
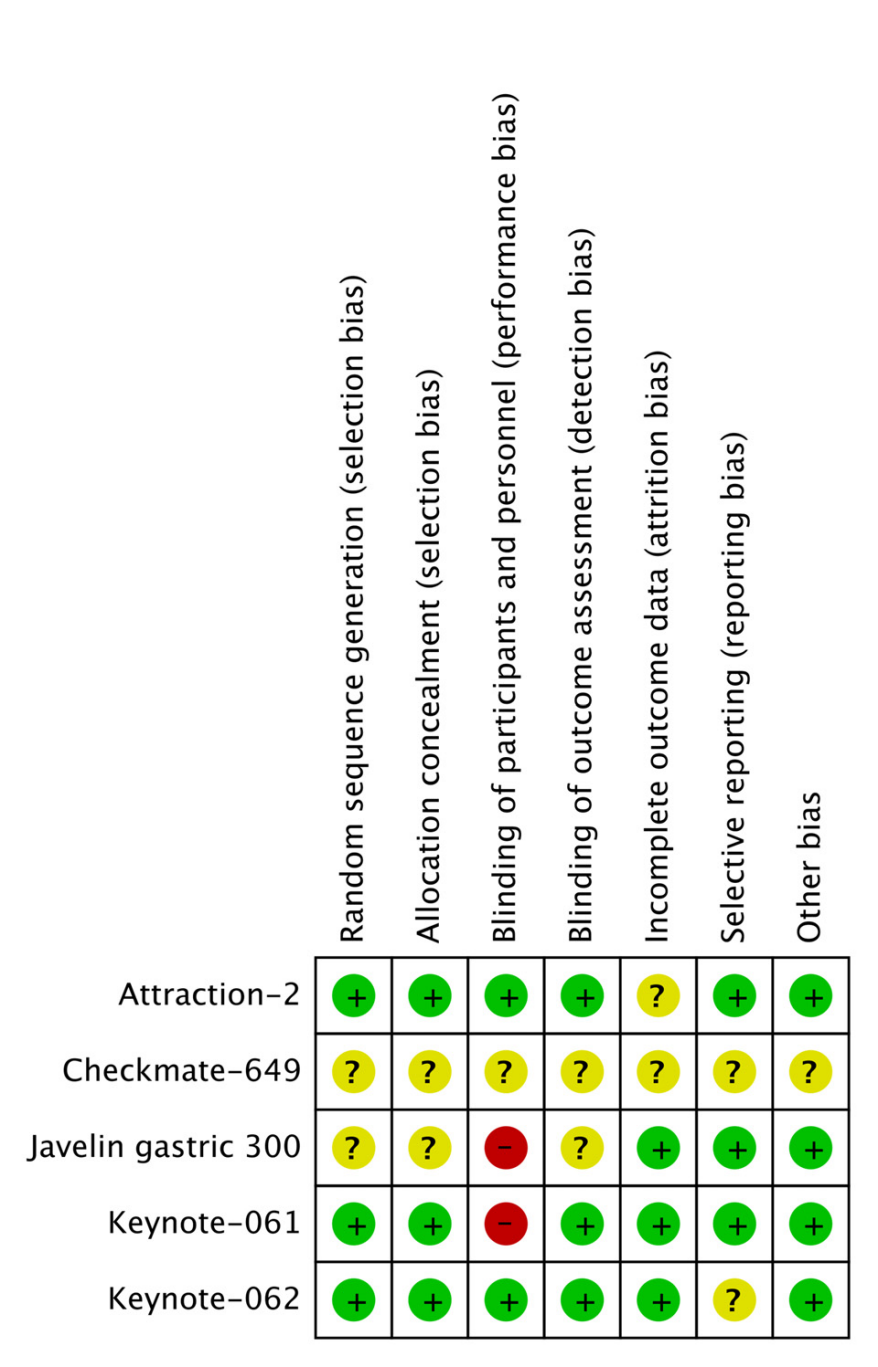
Quality assessment of included studies with full article published. Checkmate-649 and JAVELIN gastric 100 only provided meeting abstracts.

The searching diagram is presented in **Figure 1**. After the duplicates were removed and the full-text screening, 12 studies and 6,488 patients in total were finally included in our analysis (**Table 1**). All of the studies were prospective clinical trials. Six of them were phase III RCTs, and the risk of bias of included RCTs with full text is presented in **Figure 2**.

**Table 1:** Basic characteristics of included studies.

Study name	Intervention	Year	Sample size	Phase	Treatment line
KEYNOTE-062 (5)	Pembrolizumab	2020	763	III	1
KEYNOTE-659 (8) cohort 1	Pembrolizumab+SOX	2020	54	II	1
- (9)	Toripalimab+XELOX	2019	18	Ib/II	1
- (9)	Toripalimab	2019	58	Ib/II	2
KEYNOTE-059 cohort 1 (2)	Pembrolizumab	2018	259	II	≥2
KEYNOTE-059 cohort 2 (10)	Pembrolizumab+FC/XP	2019	25	II	1
KEYNOTE-059 cohort 3 (10)	Pembrolizumab	2019	31	II	1
- (11)	Camrelizumab	2019	30	I	≥2
KEYNOTE-061 (4)	Pembrolizumab	2018	592	III	2
ATTRACTION-2 (3)	Nivolumab	2017	493	III	≥3
Checkmate-032 Cohort 1 (12)	Nivolumab	2018	59	I/II	≥2
Checkmate-649	Nivolumab+XELOX/FOLFOX	2020	1,581	III	1
- (6)	Durvalumab	2020	24	Ib	2
JAVELIN Gastric 300 (13)	Avelumab	2018	371	III	≥2
JAVELIN Gastric 100 (14)	Avelumab	2020	499	III	1mn*

SOX, S-1 plus oxaliplatin; XELOX, capecitabine plus oxaliplatin; FC, 5-FU plus cisplatin; XP, capecitabine plus cisplatin.

*1mn stands for first-line maintenance.

### Response to Immunotherapy in Allover Population

**Figure 3:**
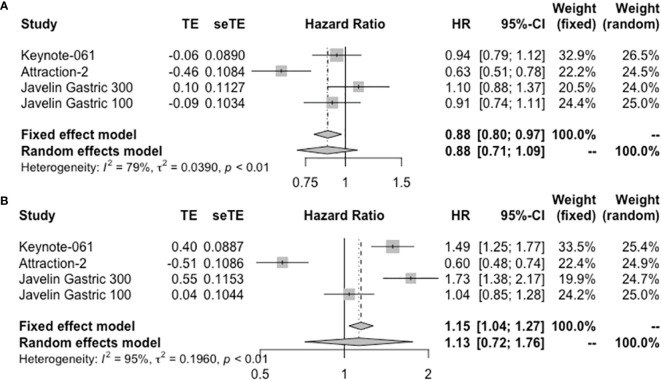
Forest plot of **(A)** OS and **(B)** PFS in allover population receiving single-agent immunotherapy. OS, overall survival; PFS, progression-free survival.

Eleven studies provided treatment outcomes of immunotherapy in allover patient population (**Table 2**). For monotherapy, OS ranged from 3.4 to 20.7 months. PFS ranged from 1.4 to 3.3 months. Durvalumab and avelumab, which are both PD-L1 inhibitors, showed the worst survival parameters and response rate among all ICIs. First-line pembrolizumab in KEYNOTE-059 cohort 2 reported the longest OS and PFS, which were superior to those other studies in second-line or later-line treatment. By integrating the data from the four RCTs, ICI monotherapy did not show superiority compared with standard care both in OS (HR = 0.88, 95% CI: 0.71–1.09) and PFS (HR = 1.13, 95% CI: 0.72–1.76) (**Figure 3**). The pooled ORR and DCR for allover population receiving single-agent immunotherapy are 10% (95% CI: 6%–15%) and 34% (95% CI: 22%–47%), respectively (**Figure S1**). After durvalumab and avelumab were removed from analysis, ORR increased to 12% (95% CI: 10%–14%) and DCR increased to 39% (95% CI: 34%–44%). On the other hand, only three studies reported the results of ICI plus chemotherapy as experimental arm in allover population. OS and PFS were similar between studies. Toripalimab plus XELOX reported an ORR of 67%, pembrolizumab+FC/XP had an ORR of 60%, and pooled ORR is 63% (**Figure S1**).

**Table 2:** Survival information immunotherapy in allover population.

Treatment	Sample size	Treatment line	OS (m)	PFS (m)
*Monotherapy*				
Toripalimab	58	2	4.8	1.9
Pembrolizumab^1^	31	1	20.7 (9.2–20.7)	3.3 (2–6)
Pembrolizumab^2^	259	≥2	5.6 (4.3–6.9)	2 (2–2.1)
Camrelizumab	30	≥2	NA	2
Pembrolizumab^3^	296	2	6.7 (5.4–8.9)	1.5 (1.4–1.6)
Nivolumab^4^	268	≥3	5.26 (4.6–6.37)	1.61 (1.54–2.3)
Nivolumab^5^	59	≥2	6.2 (3.4–12.4)	1.4 (1.2–1.5)
Durvalumab	24	2	3.4 (1.7–4.4)	1.6 (1–1.8)
Avelumab^6^	185	≥2	4.6 (3.6–5.7)	1.4 (1.4–1.5)
Avelumab^7^	249	1mn^8^	10.4 (9.1–12)	3.2 (2.8–4.1)
*Combined therapy*				
Toripalimab+XELOX	18	1	NR	5.8
Pembrolizumab+FC/XP	25	1	13.8 (8.6–NR)	6.6 (5.9–10.6)
Nivolumab+XELOX/FOLFOX	782	1	13.8 (12.6–14.6)	7.7 (7.1–8.5)

NR, not reached; NA, not available; OS, overall survival; PFS, progression-free survival; XELOX, capecitabine plus oxaliplatin; FC, 5-FU plus cisplatin; XP, capecitabine plus cisplatin; FOLFOX, fluorouracil plus oxaliplatin.

^1^Results from KEYNOTE-059 cohort 3.

^2^Results from KEYNOTE-059 cohort 1.

^3^Results from KEYNOTE-061.

^4^Results from ATTRACTION-2.

^5^Results from Checkmate-032.

^6^Results from JAVELIN Gastric 300.

^7^Results from JAVELIN Gastric 100.

^8^1mn stands for first-line maintenance.

### Outcomes of Immunotherapy at Different PD-L1 Cutoff Values

#### PD-L1-Negative Population

**Figure 4:**
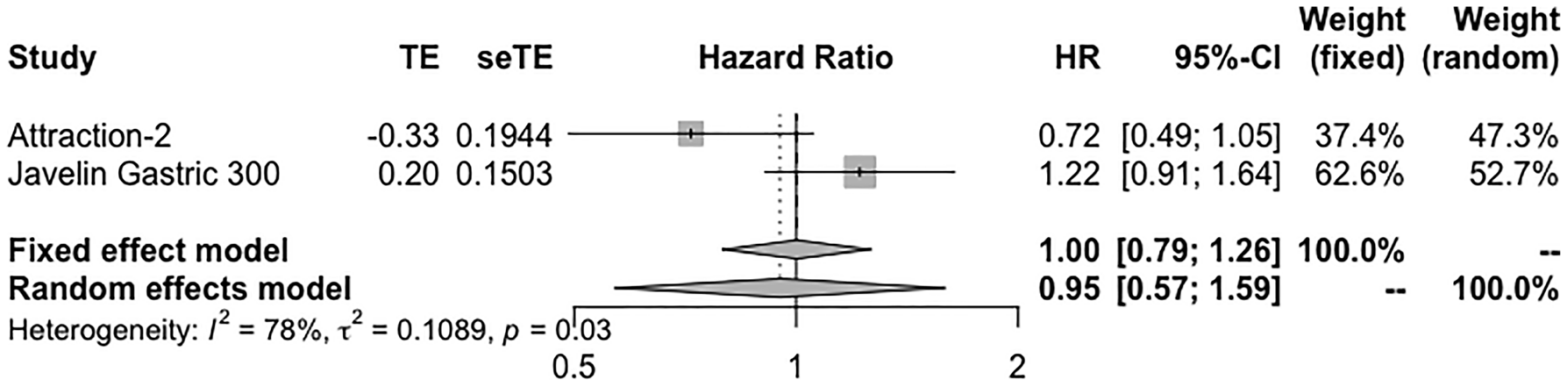
Forest plot of OS in PD-L1-negative patients receiving single-agent immunotherapy. OS, overall survival.

Only three studies reported the survival information in PD-L1-negative population receiving ICI monotherapy. Toripalimab reported an OS of 5.3 months in the second-line setting. In ATTRACTION-2, nivolumab reported an OS of 6.05 months (95% CI 4.83–8.54) in heavily treated patients. And JAVELIN Gastric 300 reported an OS of 4.6 months (95% CI: 3.4–6.3 months). PFS in toripalimab and avelumab was 1.9 and 1.4 months, respectively. Seven studies reported the results of ORR, which ranged from 0% to 26.7%; and the DCR of camrelizumab, nivolumab, and durvalumab was 53.3%, 42%, and 18.2%, respectively. The pooled ORR and DCR are 3% (95% CI: 1%–5%) and 38% (95% CI: 25%–50%), respectively (**Figure S2**). Integrated HR showed no difference in OS when comparing immunotherapy with standard treatment (HR = 0.95, 95% CI: 0.57–1.59) (**Figure 4**). Two studies reported ORR of ICI plus chemotherapy in the PD-L1-negative subgroup. Toripalimab plus XELOX exhibited an ORR of 66.7%; and in KEYNOTE-059 cohort 2, ORR was 37.5%. The pooled ORR was 57% (95% CI: 0.37–0.76) (**Figure S2**).

#### PD-L1 CPS ≥1

**Figure 5:**
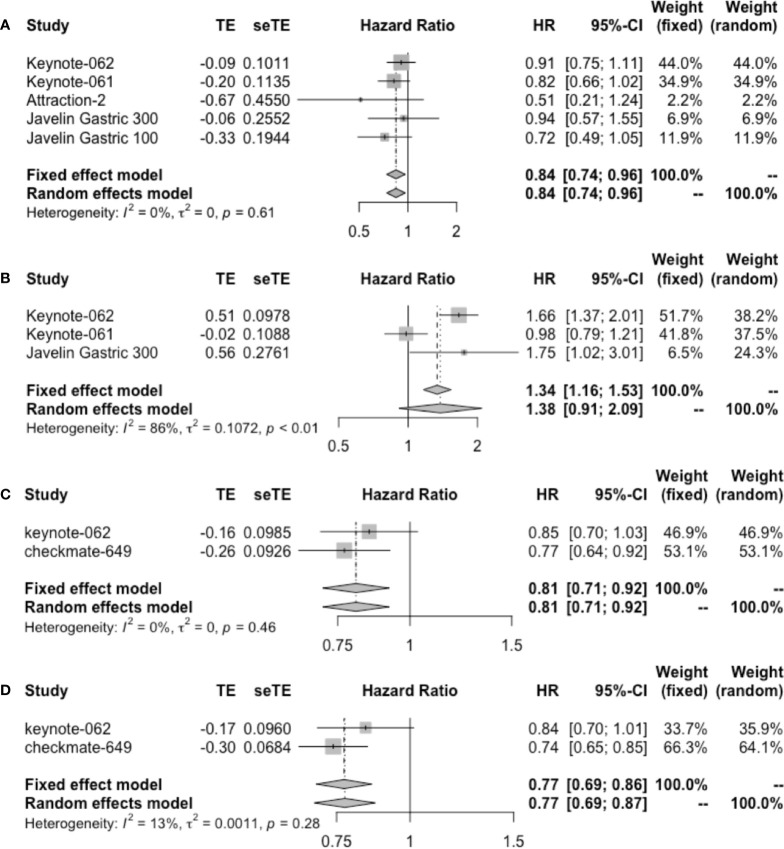
Forest plot of **(A)** OS and **(B)** PFS in patients with PD-L1 CPS ≥1 administrated with single-agent immunotherapy. **(C)** OS and **(D)** PFS in patients with PD-L1 CPS ≥1 receiving immunotherapy plus chemotherapy. OS, overall survival; PFS, progression-free survival; CPS, combined positive score.

In patients administrated with ICI monotherapy, OS ranged from 2.9 to 14.9 months, and PFS ranged from 1.4 to 5.5 months (**Table 3**). Durvalumab reported the worst OS data. Combined regimens exhibited longer survival, with OS ranging from 11.1 to 14 months and PFS ranging from 6.9 to 9.4 months. The pooled ORR for monotherapy and combined therapy is displayed in **Figure S3**, with 13% (95% CI: 8%–18%) and 48% (95% CI: 43%–54%), respectively. The DCR was 30% for single-agent therapy (**Figure S3C**), and it increased to 40% when durvalumab was removed from analysis as a PD-L1 inhibitor. Pooled HR of OS (HR = 0.84, 95% CI: 0.74–0.96) but not PFS (HR = 1.38, 95% CI: 0.91–2.09) in patients receiving monotherapy showed significance (**Figure 5**). Combined immunotherapy showed favorable OS (HR = 0.81, 95% CI: 0.71–0.92) and PFS (HR = 0.77, 95% CI: 0.69–0.86) in PD-L1 CPS ≥1 patients (**Figure 5**).

**Table 3:** Survival information of patients with PD-L1 CPS ≥1 receiving immunotherapy.

Treatment	Sample size	Treatment line	OS (m)	PFS (m)
*Monotherapy*				
Pembrolizumab^1^	256	1	10.6 (7.7–13.8)	2 (1.5–2.8)
Toripalimab	8	2	12.1	5.5
Pembrolizumab^2^	196	2	9.1 (6.2–10.7)	1.6 (1.5–2.7)
Nivolumab	16	≥3	5.22 (2.79–9.36)	NA
Durvalumab	9	2	2.9 (0.8–7)	1.7 (0.8–1.8)
Avelumab^3^	46	≥2	4 (2.5–7.6)	1.4 (1.4–2.8)
Avelumab^4^	74	1mn^7^	14.9 (8.7–17.3)	NA
*Combined therapy*				
Pembrolizumab+FC/XP^5^	257	1	12.5 (10.8–13.9)	6.9 (5.7–7)
Pembrolizumab+SOX	54	1	NR	9.4 (6.6–NR)
Pembrolizumab+FC/XP^6^	16	1	11.1 (5.4–22.3)	NA
Nivolumab+XELOX/FOLFOX	641	1	14 (12.6–15)	7.5 (7–8.4)

NR, not reached; NA, not available; OS, overall survival; PFS, progression-free survival; FC, 5-FU plus cisplatin; XP, capecitabine plus cisplatin; SOX, S-1 plus oxaliplatin; XELOX, capecitabine plus oxaliplatin; FOLFOX, fluorouracil plus oxaliplatin.

^1^Results from KEYNOTE-062.

^2^Results from KEYNOTE-061.

^5^Results from KEYNOTE-062.

^6^Results from KEYNOTE-059 cohort 2.

^7^1mn stands for first-line maintenance.

The efficacy of toripalimab was compared between PD-L1-positive and PD-L1-negative groups. Although PD-L1-positive patients showed higher ORR (37.5% *vs.* 8.5%), there was no difference in OS (*p* = 0.45) and PFS (HR = 0.53, 95% CI: 0.25–1.11; *p* = 0.092). Durvalumab also reported no differences between PD-L1-positive and PD-L1-negative subgroups receiving ICI therapy.

#### PD-L1 CPS ≥5

Two studies reported the results of ICI monotherapy in PD-L1 CPS ≥5 patients. In KEYNOTE-061, pembrolizumab exhibited an OS of 10.4 months (95% CI: 6.7–15.5) and PFS of 1.6 months (95% CI: 1.4–2.8), the ORR and DCR were 20% and 44.2%, respectively. Camrelizumab reported ORR and DCR of 20% and 40%, respectively. Moreover, camrelizumab compared the ORR between PD-L1 CPS <5 and ≥5 groups, and there was no significant difference. As for combined therapy, Checkmate-649 reported a great superiority of nivolumab plus chemotherapy over standard care; the OS was 14.4 months (95% CI: 13.1–16.2; HR = 0.71, 95% CI: 0.59–0.86) and PFS was 7.7 months (95% CI: 7–9.2; HR = 0.68, 95% CI: 0.56–0.81); the ORR and DCR were 60% and 88%, respectively.

#### PD-L1 CPS ≥10

In the PD-L1 CPS ≥10 subgroup, the efficacy of only pembrolizumab as an ICI was explored; results are summarized in **Table 4**. Pembrolizumab monotherapy in KEYNOTE-062 showed the longest OS of 17.4 months; in KEYNOTE-059 cohort 3, pembrolizumab exhibited the worst survival outcomes of 7.9 months. Pooled ORR is displayed in **Figure S4**. ORR in patients receiving ICI monotherapy and combined therapy was 23% (95% CI: 17%–29%) and 58% (95% CI: 49%–66%), respectively. Only KEYNOTE-061 reported the DCR of 47.1%.

**Table 4:** Results of immunotherapy in patients with PD-L1 CPS ≥10.

Treatment	Sample size	Treatment line	OS (m)	PFS (m)
Pembrolizumab^1^	92	1	17.4 (9.1–23.1)	2.9 (1.6–5.4)
Pembrolizumab^2^	46	1	7.9 (5.8–11.1)	2.1
Pembrolizumab^3^	53	2	10.4 (5.9–17.3)	2.7
Pembrolizumab+FC/XP	99	1	12.3 (9.5–14.8)	NA
Pembrolizumab+SOX	31	1	NA	8.1 (5.5–NR)

NA, not available; OS, overall survival; PFS, progression-free survival; FC, 5-FU plus cisplatin; XP, capecitabine plus cisplatin; SOX, S-1 plus oxaliplatin.

^1^Results from KEYNOTE-062.

^2^Results from KEYNOTE-059 cohort 1.

^3^Results from KEYNOTE-061.

## Discussion

Following the publication of several clinical trials, immunotherapy transformed the treatment of gastric cancer, and PD-L1 has been proposed as a biomarker for gastric cancer immunotherapy. Here, we primarily summarized the newest studies that reported the results of subgroup analysis according to PD-L1 expression, and we found that PD-L1 CPS could predict the efficacy of immunotherapy, especially for single-agent therapy.

Although ICI was demonstrated to have definite efficacy in gastric cancer palliative care, ICI monotherapy exhibited a relatively low response rate, with pooled ORR of 10% in allover population. However, we found that the pooled ORR in PD-L1-negative, PD-L1 CPS ≥1, PD-L1 CPS ≥5, and PD-L1 CPS ≥10 population was 3%, 13%, 20%, and 23%, respectively. The ORR increased with gastric cancer PD-L1 CPS. As for survival, after integrating RCTs that reported the results of HR in patients with PD-L1 CPS ≥1 subgroup, we found borderline positive results of single-agent immunotherapy in gastric cancer, with 95% CI ranging from 0.74 to 0.96 in OS, while the pooled results in both allover and PD-L1 CPS <1 population were negative. Although the results of ICI monotherapy in PD-L1 CPS ≥1 patients may be unstable, ICI monotherapy should not be recommended to PD-L1-negative patients due to the low response rate and scarce survival advantage. Interestingly, from another perspective, the range of OS in PD-L1 CPS ≥10 patients did not overlap with that in PD-L1-negative patients, which reflected the definite survival advantage. On the other hand, the range of PFS resembled each other at different PD-L1 cutoff values. It is hard to explain the different impact of immunotherapy to PFS and OS; and the relationship among ORR, PFS, and OS in immunotherapy may differ from that in chemotherapy and needs further confirmation. Among all the studies, toripalimab and durvalumab reported comparative results of efficacy and survival between PD-1-positive and PD-1-negative groups in the same cohort. PD-L1-positive patients showed higher ORR (37.5% *vs.* 8.5%) in patients administrated with toripalimab, while both toripalimab and durvalumab had no significant survival difference between PD-L1-positive and PD-L1-negative groups (9, 15). As the sample size of toripalimab study was relatively small and no patients showed objective response when treated with durvalumab, we should make a judgment more carefully.

On the other hand, when ICI was combined with chemotherapy, the correlation between PD-L1 expression and ORR was not obvious. The pooled ORR in PD-L1-negative, PD-L1 CPS ≥1, PD-L1 CPS ≥5, and PD-L1 CPS ≥10 population was 57%, 48%, 60%, and 58%, respectively. It seems that when ICI was combined with chemotherapy, the improvement owing to the increase of PD-L1 expression was not prominent. Also, the range of PFS and OS overlapped among different subgroups. What is for sure is that the ORR was improved remarkably after ICI was combined with chemotherapy. Despite the failure to improve OS in KEYNOTE-062, the ORR in the pembrolizumab plus chemotherapy arm was 48.6% among PD-L1 CPS ≥1 patients, while pembrolizumab monotherapy only got 14.8% (5). As for survival, pooled data from Checkmate-649 and KEYNOTE-062 showed significant OS benefit, with an HR of 0.81 (95%: 0.71–0.92), which may suggest an option for ICI plus chemotherapy in PD-L1 CPS ≥1 patients (5, 6). Also, other ICIs also reported favorable response to combined immunotherapy in gastric cancer, especially in the first-line setting (7, 11, 16).

Apart from efficacy of ICI at different PD-L1 cutoff values, we found that first-line ICI monotherapy had better outcome than second-line and later-line treatment. The phenomenon may due to the intact immune microenvironment and reserved bone marrow function (17, 18). As the antitumor activity of ICI relies on the preexisting immune cells in the tumor tissue, in heavily treated patients, the previous adoption of chemotherapy could destroy the tumor immune microenvironment. Also, as treatment goes beyond the second or later line, the function of bone marrow as well as the immune cells could also be affected. It has been reported that immunotherapy showed a lower efficacy rate in patents with poor performance status (19).

Different ICI types could also make a difference on the treatment outcomes. What is notable in our investigation is the low response rate and survival outcomes in PD-L1 inhibitors, such as avelumab and durvalumab; few patient benefited from these drugs (13). However, in non-small cell lung cancer, things are different. PD-L1 inhibitors, for example, atezolizumab also showed long-lasting response to tumor cells (20). The disorganized PD-L1 inhibitors may due to the different tumor microenvironment of gastric cancer and other tumors, while the efficacy of PD-L1 inhibitors plus chemotherapy to gastric cancer was still unknown. As we all know, nivolumab and pembrolizumab are two PD-1 inhibitors that both exhibited powerful antitumor effects across diverse tumor types. However, the results of the two drugs in gastric cancer differ dramatically. Although PD-1 inhibitors target the same site, we should treat them individually.

There were several limitations in our study. Although we enrolled the newest clinical trials across databases comprehensively, the data in some subgroups were still lacking. For example, few study reported the data on PD-L1 CPS ≥10; most of the studies only stratified patients into PD-L1-positive and PD-L1-negative groups. Also, there is still no standard PD-L1 testing kit for clinical practice currently; the IHC kit in different clinical trials varied. For pembrolizumab, 22c3 was widely used, while for nivolumab, 28-8 was adopted. However, previous studies reported a highly consistent testing result; in consideration of this, we pooled the results according to the expression of PD-L1 across different studies (21, 22).

In conclusion, our study summarized the current clinical trials in gastric cancer immunotherapy that provided subgroup results according to PD-L1 expression. ICI monotherapy significantly improved OS in PD-L1 CPS ≥1 or higher population but was not recommended for PD-L1-negative patients due to an extremely low response rate. ICI plus chemotherapy exhibited a favorable response rate in allover gastric cancer patients irrespective of PD-L1 expression. PFS and OS were prolonged by combined immunotherapy in PD-L1-positive patients.

## Data Availability Statement

The original contributions presented in the study are included in the article/**Supplementary Material**. Further inquiries can be directed to the corresponding author.

## Author Contributions

ZP designed this project. TX as the first author summarized and wrote this paper. ZZ participated in the searching and screening process. XZ and CQ gave advice on statistics and paper architecture. LS polished this paper. All authors contributed to the article and approved the submitted version.

## Funding

The third round of public welfare development and reform pilot projects of Beijing Municipal Medical Research Institutes (Beijing Medical Research Institute, 2019-1), National Natural Science Foundation of China (No. 81902514) and Clinical Medicine Plus X—Young Scholars Project of Peking University and the Fundamental Research Funds for the Central Universities (No. PKU2021LCXQ016).

## Conflict of Interest

The authors declare that the research was conducted in the absence of any commercial or financial relationships that could be construed as a potential conflict of interest.

## Publisher’s Note

All claims expressed in this article are solely those of the authors and do not necessarily represent those of their affiliated organizations, or those of the publisher, the editors and the reviewers. Any product that may be evaluated in this article, or claim that may be made by its manufacturer, is not guaranteed or endorsed by the publisher.
