# Supplementary material for: Appropriate PD-L1 Cutoff Value for Gastric Cancer Immunotherapy: A Systematic Review and Meta-Analysis
Source: Front Oncol. 2021 Sep 1;11:646355. doi: 10.3389/fonc.2021.646355 (PMC8440909; doi:10.3389/fonc.2021.646355)
Supplement: Supplementary file 1 [file DataSheet_1.docx]

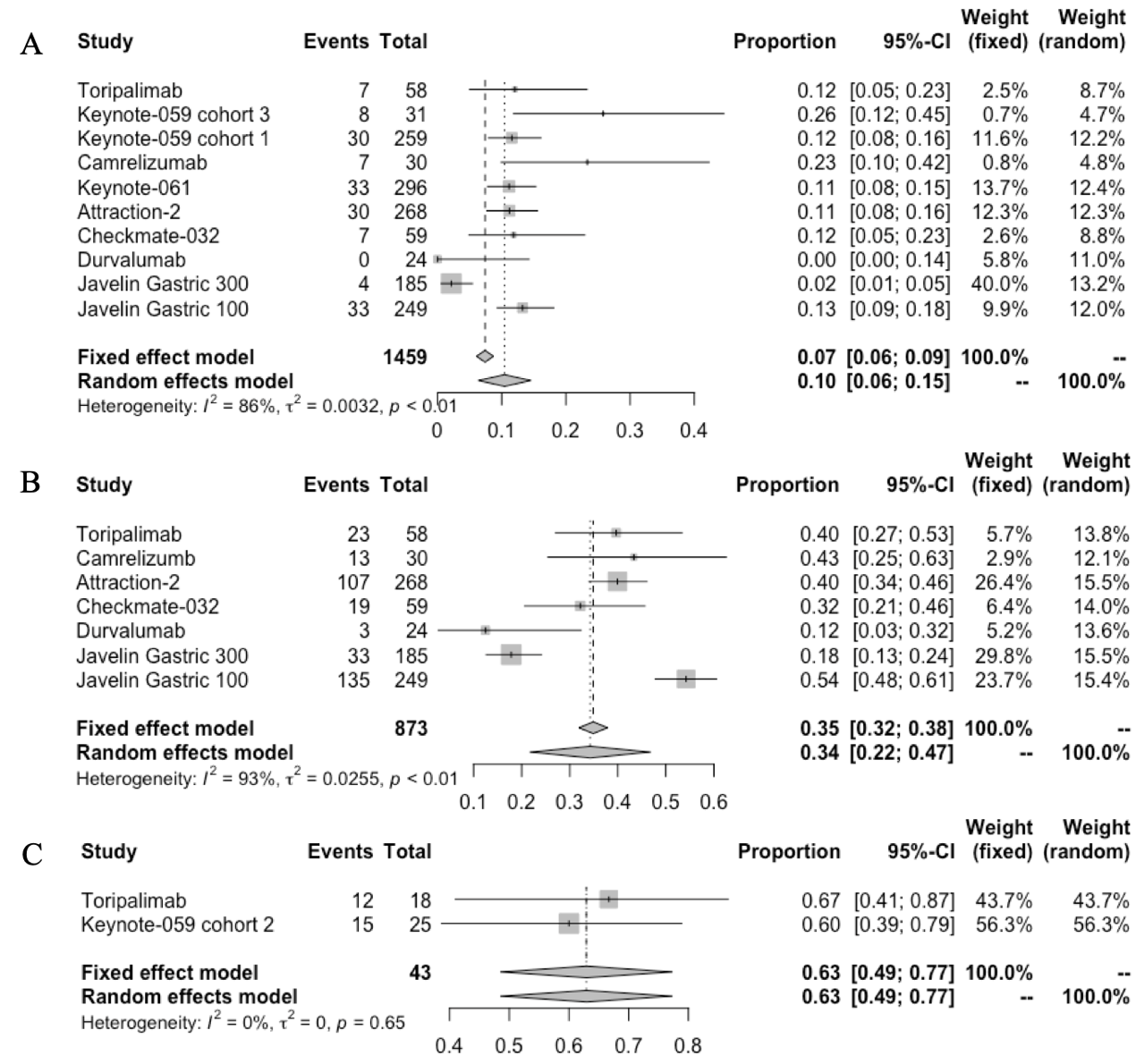


Figure S1: Forest plot of (A) ORR and (B) DCR in all-over population receiving single agent immunotherapy. (C) ORR of all-over population receiving combined immunotherapy.


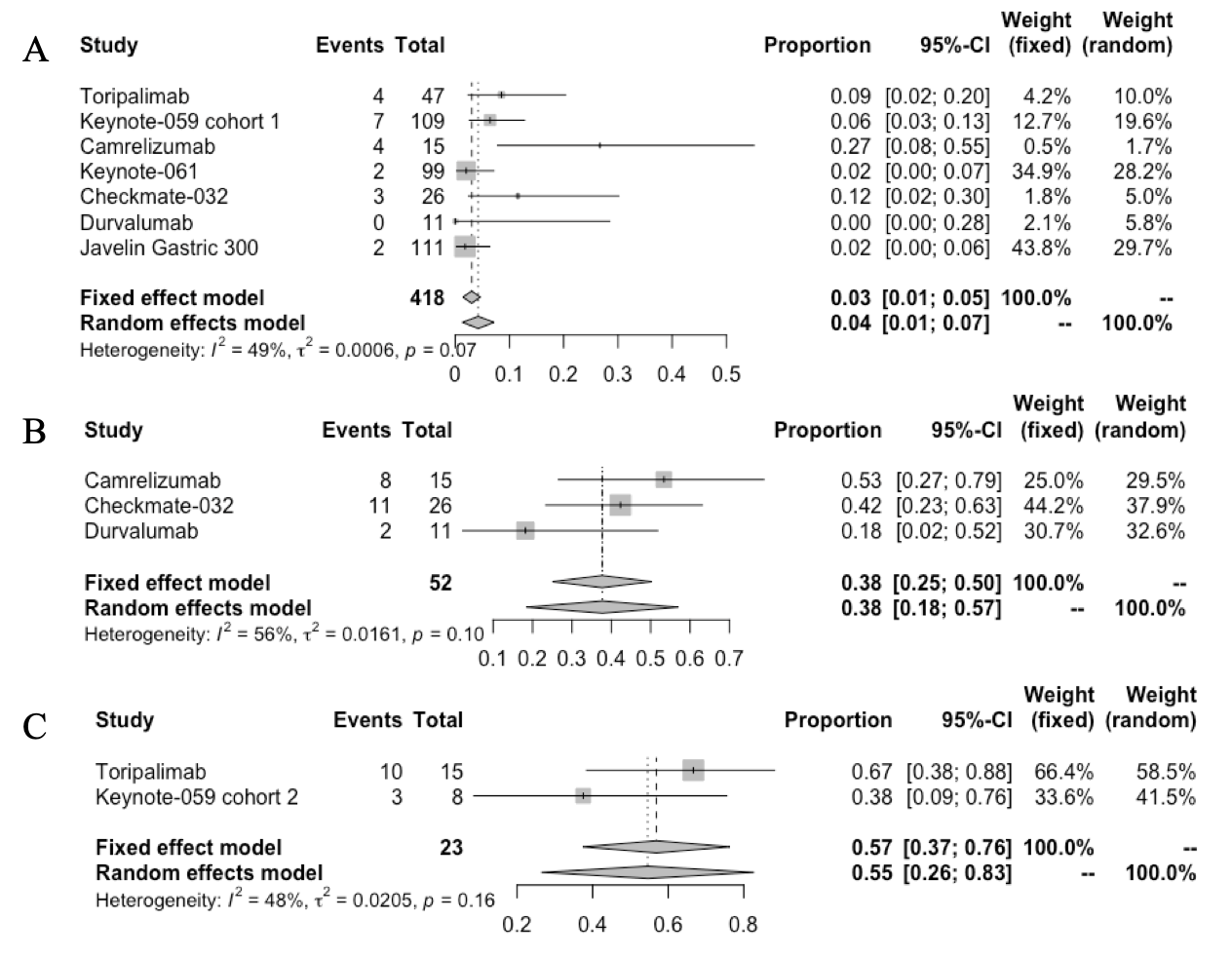


Figure S2: Forest plot of (A) ORR and (B) DCR in PD-L1 negative population receiving single agent immunotherapy. (C) Forest plot of ORR in PD-L1 negative population receiving combined immunotherapy.


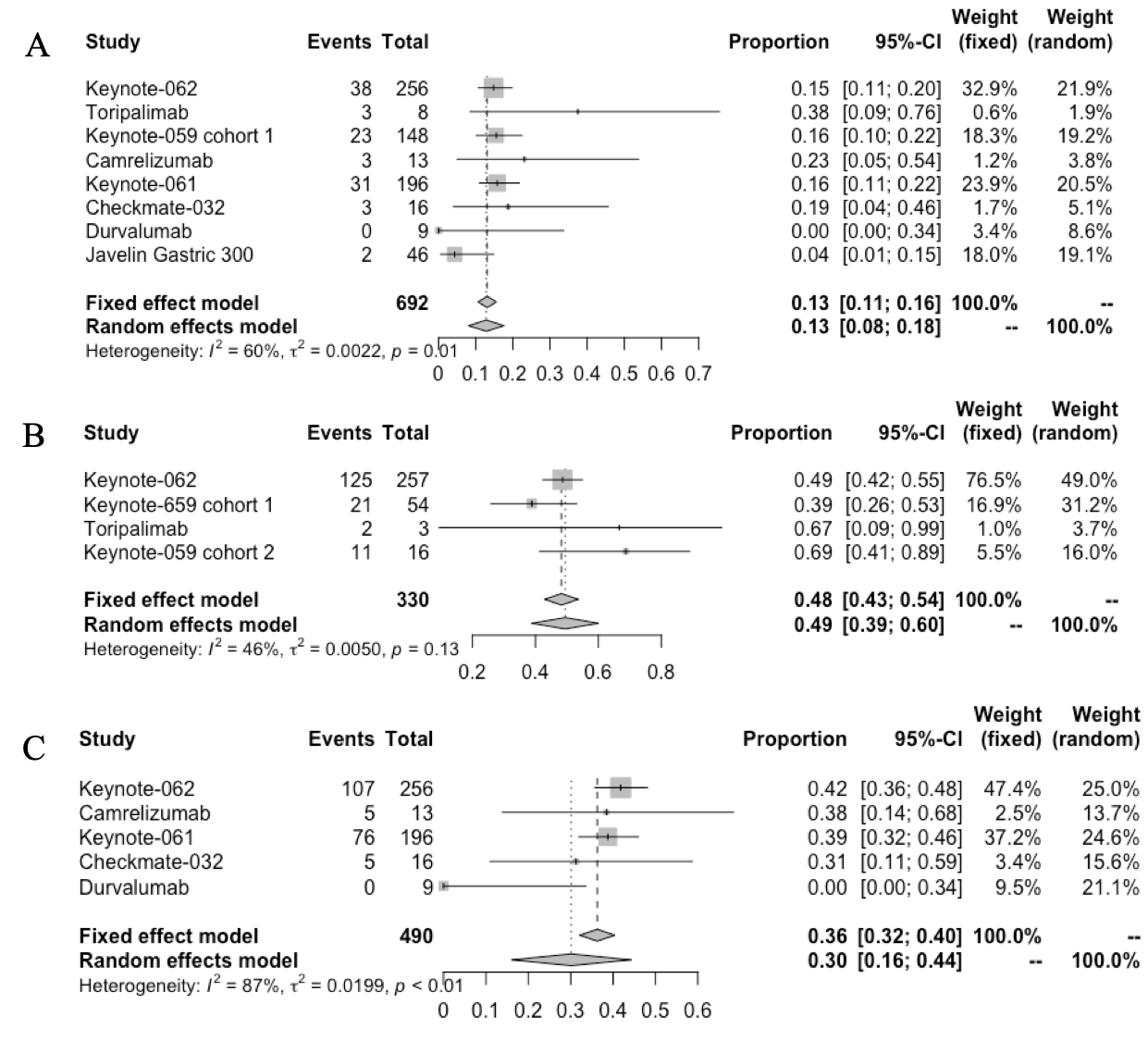


Figure S3: Forest plot of ORR in patients with PD-L1 CPS ≥ 1 receiving (A) ICI monotherapy or (B) combined immunotherapy.


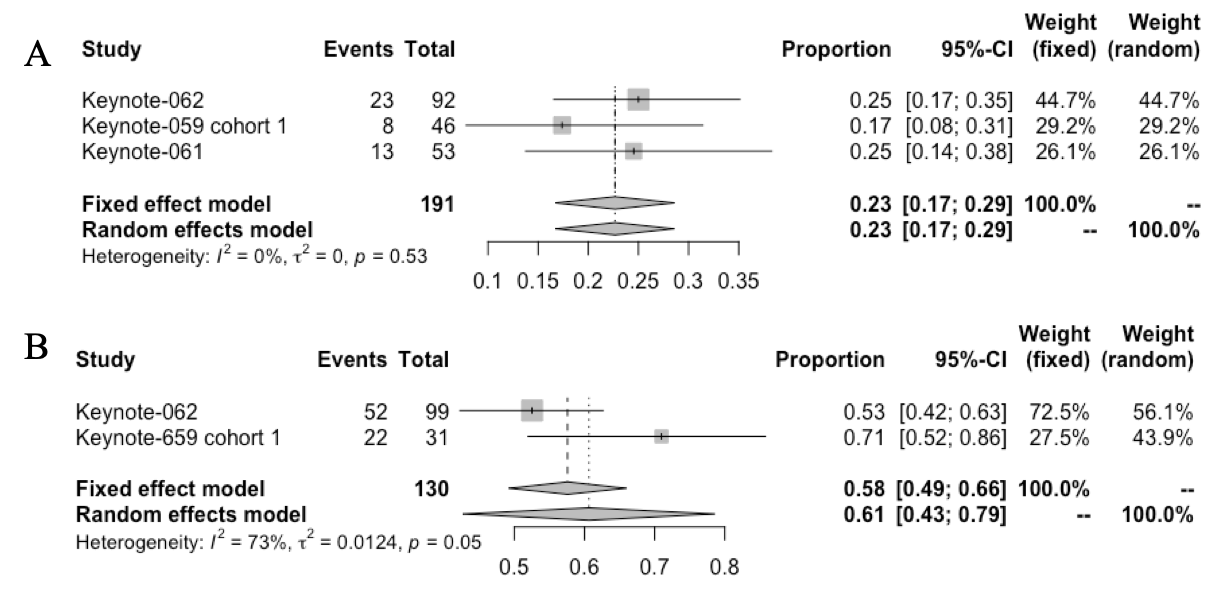


Figure S4: Forest plot of ORR in patients with PD-L1 CPS ≥10 receiving (A) single agent immunotherapy or (B) combined immunotherapy.
